# Clinical and prognostic pan-cancer analysis of m6A RNA methylation regulators in four types of endocrine system tumors

**DOI:** 10.18632/aging.104064

**Published:** 2020-11-20

**Authors:** Kai Li, Haiqing Luo, Hui Luo, Xiao Zhu

**Affiliations:** 1 Guangdong Key Laboratory for Research and Development of Natural Drugs, The Marine Biomedical Research Institute, Guangdong Medical University, Zhanjiang 524023, Guangdong, China; 2 The Marine Biomedical Research Institute of Guangdong Zhanjiang, Zhanjiang 524023, Guangdong, China; 3 Southern Marine Science and Engineering Guangdong Laboratory Zhanjiang, Zhanjiang 524023, Guangdong, China; 4 Cancer Center, Affiliated Hospital, Guangdong Medical University, Zhanjiang 24023, Guangdong, China

**Keywords:** endocrine system tumors, m6A methylation regulators, pan-cancer analysis, prognosis, risk score

## Abstract

N6-methyladenosine (m6A), internal modification of mRNA, has recently been reported to be an important regulatory mechanism affecting tumor proliferation. However, its role in endocrine system tumors is poorly understood. We obtained datasets for four types tumors from the TCGA database, analyzed the GTEx database as a supplement to the control group, and used “Perl” and “R” software to analyze the datasets. Then we differentiated the expression level, used it to cluster consensus. Besides, we established lasso regression model to screen variables, used univariate and multivariate cox analyses to explore the independent risk factors associated with cancer prognosis. The results indicated that except for WTAP, the expression level of METTL3 was negatively correlated with other genes. The expression level of WTAP and METTL16 was positively correlated with overall survival (OS). Moreover, we found that different clinical subtypes of adrenal cortical carcinoma had significant differences in survival status, histologic grading, pathological T grade, and OS. Furthermore, different clinical subtypes of thyroid carcinoma had significant differences in histologic grading and pathological T grade. The differential expression of m6A regulatory genes is closely associated with the presence of endocrine-system-related tumors, and risk scores can be used to assess prognosis.

## INTRODUCTION

According to the recent data, the incidence of endocrine system tumors is increasing year by year, although the proportion is not large which cannot be ignored [1]. Despite advances in the diagnosis and treatment of endocrine tumors (adrenal cortical carcinoma (ACC), pheochromocytoma and paraganglioma (PCPG), thyroid carcinoma (THCA) and thymoma (THYM)) in recent decades, some patients still have a poor prognosis, especially ACC [2]. Recently, a number of studies have found that m6A regulatory genes play important roles in the occurrence and development of tumors [3], which also include the endocrine system tumors [4].

N6-methyladenosine (m6A), also known as RNA methylation modification, occurs on the sixth nitrogen atom (N) of adenine (A) and is enriched in mRNA [5]. It is mainly distributed in the coding sequence and 3'UTR region of mRNA. The modification process is dynamic and reversible, involving three parts: writers, erasers and readers [6, 7]. Writers (METTL3, METTL14, METTL16, WTAP, RBM15, RBM15, KIAA1429, CBLL1, ZC3H13) are involved in the methylation process, and erasers (FTO, ALKBH5) are involved in the demethylation process. Readers (YTHDF1, YTHDF2, YTHDF3, YTHDC1, YTHDC2, HNRNPA2B1, HNRNPC, IGF2BP1, IGF2BP2, IGF2BP3, ZNF217, RBMX) identify RNA methylation modification of information, and participate in the process of translation, degradation of RNA downstream. Regulatory gene interactions affect m6A regulation [8]. For example, CircNSUN2, which is formed by mRNA modification, can bind to intracellular m6A reader YTHDC1 and be regulated by YTHDC1 to determine the nuclear localization. When removed to the cytoplasm, CircNSUN2 bind to reader IGF2BP2, combine with downstream HMGA2 mRNA to improve the stability of HMGA2 mRNA, and ultimately promote liver metastasis of colorectal cancer tumors [9].

M6A is widely involved in cell proliferation and differentiation, immunity, tumor generation and metastasis, and other life processes. Its poor regulation affects the normal life process, resulting in decreased cell proliferation, immune changes and cell carcinogenesis [10, 11]. Previous studies have found that m6A regulates genes and plays a key role in blood development [12]. Peng et al. 's study found that inhibition of the FTO-FOXO1 pathway resulted in weight loss and decreased blood sugar, which could be considered for the treatment of the metabolic syndrome [13]. M6A gene regulation is closely related to cell proliferation and malignant transformation, especially the METTL3 which recruited downstream translation initiation factors by identifying GGAC sequences, rather than by affecting methylation pathways, to affect translation, thus increasing the expression of oncogenes and affecting the occurrence of cancer [14, 15]. A study has shown that METTL3 is highly expressed in NSCLC tissues, and the expression level of METTL3 is positively correlated with the expression of MiR-33a in NSCLC tissues [16]. METTL3 promotes the development of colorectal cancer [17], gastric cancer [18], bladder cancer [19], breast cancer [20], renal cell carcinoma [21], and pancreatic cancer cells [22], and the occurrence of non-small cell lung cancer is related to METTL3 [23]. The decrease of the METTL3 level can promote the apoptosis of cancer cells through MiR-33a. Previous studies have reported that changing the expression of the METTL3 gene to increase the m6A regulatory genes level in U251 cells affects the occurrence and development of glioma [24]. Moreover, many studies have indicated that m6A regulatory gene greatly affects tumor proliferation. Kwok et al. found that mutations in m6A regulatory gene are closely related to the presence of TP53 mutations in acute myeloid leukemia patients [25]. A study of Ji et al. showed that patients with prostate cancer can be found to have high expression of reader protein and methyltransferase complex and poor survival benefit [26]. Tang et al. suggested that m6A RNA methylation regulator plays a critical role in the potential malignant progression and prognostic value of uveal melanoma [27]. The study of Meng et al. indicated that overall survival was worse in pancreatic cancer patients with m6A regulatory gene change [28]. However, little is known about the relationship between m6A-related genes and endocrine tumors. We investigated and evaluated different clinical features of m6A regulating tumor-related genes, and found the relationship between the expression changes and the clinical pathology of endocrine system tumors, including survival, provide a reference for subsequent research guidance value.

## RESULTS

### The expression of m6A regulatory genes is related to endocrine system tumors

**Figure 1:**
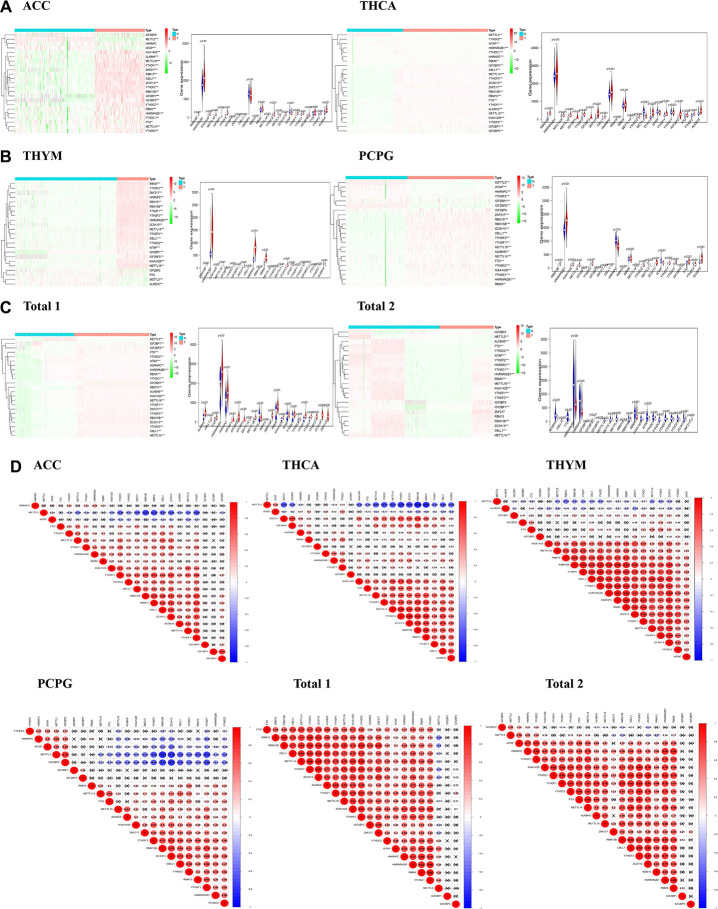
**The distribution of m6A regulatory genes in endocrine system tumors.** (**A**–**C**) Heatmaps defined by the expression level of 23 m6A regulatory genes (red for up-regulated, green for down-regulated and the tree on the left shows the clustering results of different genes in different samples) and vioplots visualized the differential m6A regulatory genes in endocrine system tumors (assuming blue is normal tissue and red is tumors); (**D**) Correlation analysis of expression of 23 m6A regulatory genes in endocrine system tumors.

In view of the important role of m6A regulatory genes plays in the development of tumors, we systematically studied the relationship between 23 m6A regulatory genes and each endocrine system tumor. The expression level of each m6A regulatory gene in endocrine system tumors was presented in the form of a heat map. The results show that, except for HNRNPC gene, the expression level of other m6A regulatory genes in ACC (Figure 1A) was distinct from that in normal tissues. Except for the IGF2BP3 gene, the expression of m6A regulatory genes in PCPG (Figure 1B) generally differed from normal tissue. The expression of 23 m6A regulatory genes in THCA (Figure 1A) was generally different from that in normal tissue. Except for IGF2BP2 and FTO gene, the expression of other m6A regulatory genes in THYM (Figure 1B) was generally distinct from normal tissue.

Then, due to different clinical variables, we divided endocrine system tumors into “Total 1” (ACC and THCA datasets) and “Total 2” (PCPG and THYM datasets). The expression level of m6A regulatory genes of each “Total” was analyzed. In the dataset of “Total 1” (Figure 1C), 23 genes were found to be different from normal tissue. In the dataset of “Total 2” (Figure 1C), except for ZNF217, the expression of m6A regulatory genes is generally different from that of normal tissue. These results indicate that most of the expression of m6A regulatory genes are associated with endocrine tumors.

We then analyzed the correlation of 23 gene expressions in tumors. Our study found that most of expression level of the genes are negatively correlated with METTL3 expression, but there are some exceptions. With the expression level of WTAP is elevated, METTL3 expression is in high level. This can be observed in each dataset (Figure 1D). These datasets illustrate the interaction of gene expression level between m6A regulatory genes.

### Consensus clustering method for m6A regulators identified two clusters of endocrine system tumors

**Figure 2:**
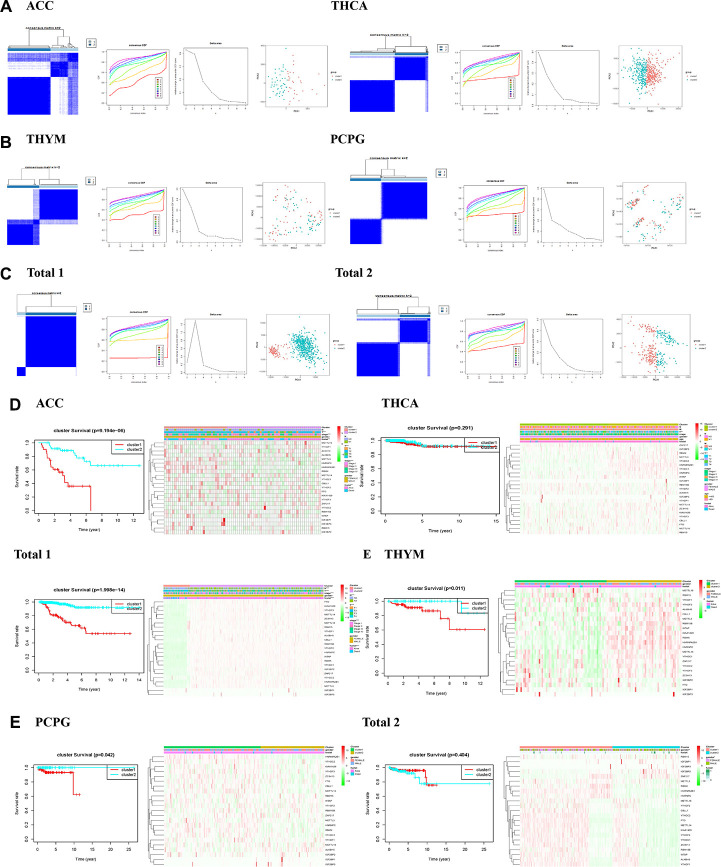
**Identification of consensus clusters by m6A RNA methylation regulators in endocrine system tumors.** (**A**–**C**) Consensus clustering matrix for k = 2, and consensus clustering cumulative distribution function (CDF) for k = 2 to 9, and relative change in area under CDF curve for k = 2–9, and principal component analysis (PCA) of total RNA expression profiles in tumors data from the cancer genome atlas (TCGA) dataset; (**D**, **E**) Heatmaps, and Kaplan–Meier overall survival (OS) curve and clinicopathologic features of two clusters defined by consistent expression of the m6A regulatory genes (clusters1/2).

In the view of the result that most of the expression of m6A regulatory genes are associated with endocrine tumors, we further investigated the relationship between m6A regulatory genes and the clinicopathological characteristics of the tumors. Clustering classification was performed based on the expression correlation of the m6A RNA methylation regulators, and the clustering stability was increased from k = 2 to 10. The clinicopathological features of the two subgroups clustered were cluster 1 and cluster 2 according to k=2 in ACC datasets (Figure 2A). We further used principal component analysis (PCA) to compare the expression curves between cluster 1 and cluster 2 subgroups, and found that there were significant differences between subgroups (Figure 2A). It is showed that the cluster 1 subgroup (Figure 2D) was significantly correlated with better survival status (P < 0.01), higher who grade (P < 0.01), and higher T status (P < 0.01). The cluster 2 subgroup was associated with worse survival status, lower who grade (P < 0.01), and lower T degree (P < 0.01). In addition, we observed that the overall survival rate (OS) of the cluster 1 subgroup was significantly shorter than that of the cluster 2 subgroup (Figure 2D). We then clustered the other tumors in the same way, observed some similar results and other differences. According to the THCA dataset, we observed no significant difference in OS (Figure 2D) between the cluster 1 subgroup and the cluster 2 subgroup, and it may be necessary to observe the survival in patients with the disease for a longer time. Besides, if only from the aspects of living condition and gender analysis PCPG and THYM datasets (Figure 2B, E), we found no significant difference of clinical features.

Then we conducted a cluster analysis on “Total”. Due to incomplete information of age and tumor metastasis in ACC clinical variables, these two variables will not be analyzed. Both the clinicopathological characteristics of cluster 1 and cluster 2 subgroups clustered by k=2 (Figure 2C), principal component analysis (PCA) was further used to compare the expression curves between cluster 1 and cluster 2 subgroups (Figure 2C). we found that the cluster 1 subgroup was associated with lower survival status (P < 0.01), higher who grade (P < 0.01), greater T (P < 0.01), and more lymph node metastasis (P < 0.01) in “Total 1” (Figure 2D). We observed cluster 1 subgroup of OS is not different from cluster 2 subgroup, only from the aspects of living condition and gender analysis, found the cluster 1 subgroup and gender (P < 0.01) in “Total 2” (Figure 2E). In general, the results showed that the expressions of m6A regulatory genes were closely related to the clinicopathological characteristics of the tumors.

### The prognostic role of m6A regulators in endocrine system tumors

**Figure 3:**
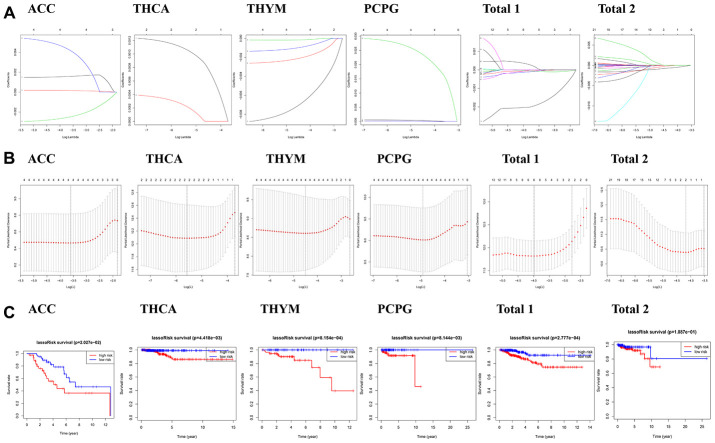
**Risk signatures with three m6A RNA methylation regulators in endocrine system tumors.** (**A**–**C**) The coefficients calculated by multivariate Cox regression using LASSO are shown, and Kaplan–Meier overall survival (OS) curve of patients was divided into high-risk and low-risk groups according to the risk score.

In order to evaluate the prognostic effect of the m6A regulatory genes in tumors, we performed univariate Cox regression analysis on the expression level of m6A regulatory genes in the dataset. The results showed that IGF2BP1, HNRNPA2B1, METTL14 and IGF2BP3 were significantly correlated with OS of ACC patients (Figure 3A). METTL14 was the protective gene for HR = 1, and IGF2BP1, HNRNPA2B1 and IGF2BP3 were the risk genes for HR = 1. For genes with prognostic values in PCPG, RBMX, HNRNPC, IGF2BP3 and HNRNPA2B1 were significantly correlated with the OS of PCPG patients (Figure 3B), and were all risk genes with HR = 1. For genes with prognostic value in THCA, YTHDF3 and FTO were significantly correlated with OS of THCA patients (Figure 3A), and were all risk genes with HR = 1. For genes with prognostic values in THYM, CBLL1, RBM15B, KIAA1429, WTAP were significantly correlated with OS of THYM patients (Figure 3B), all of which were HR = 1 protective genes.

We also analyzed the prognostic value of each m6A RNA methylation regulator in “Total 1” (Figure 3C). The results showed that among the 23 tested genes, METTL14, WTAP, RBM15B, METTL16, FTO and IGF2BP1 were significantly correlated with OS (P <0.05). Among the six genes, IGF2BP1 is the risk gene of HR = 1, METTL14, WTAP, RBM15B, METTL16 and FTO are the protective genes of HR = 1. In “Total 2” (Figure 3C), WTAP and METTL16 were significantly correlated with OS (P<0.05), and they were both protective genes of HR = 1. In general, WTAP, METTL16 and IGF2BP3 had a significant influence in the occurrence and development of endocrine system tumors. The reduction expression of WTAP and METTL16 can cause poorer survival in endocrine system tumors. IGF2BP3 is danger genes.

### The prognostic risk score is closely related to the clinical characteristics of endocrine system tumors

Based on the above data analysis results that m6A regulated gene expression was closely related to the clinical characteristics and prognosis of tumors, we further studied the relationship between prognostic risk score and endocrine tumors. To better determine the clinical outcome of endocrine system tumors using m6A regulatory genes, we applied the least absolute shrinkage and selection operator (LASSO) Cox regression algorithm to 23 prognostic genes in the TCGA dataset, obtained genes significantly associated with prognosis to establish risk characteristics based on the minimum criteria, and obtained the coefficients from the LASSO algorithm to calculate the risk scores of the validation dataset. To investigate the effect of genetic risk on prognosis, patients with endocrine system tumors in the dataset were separated into the low-risk group and the high-risk group according to the median risk scores. The significant differences in OS between the two groups were observed (P<0.05).

**Figure 4:**
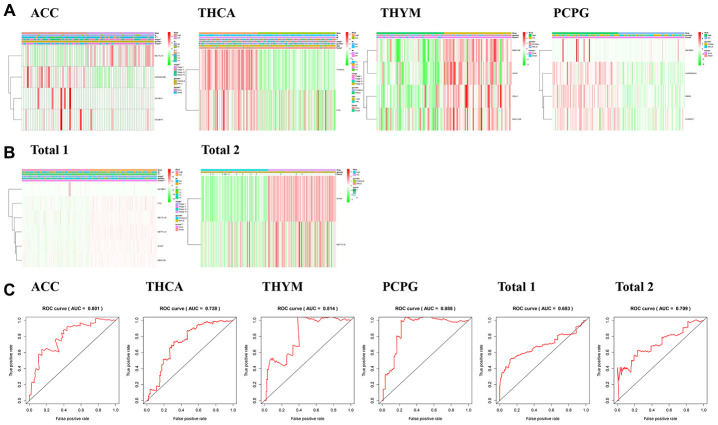
**Relationship between risk score, m6A regulatory genes and clinicopathologic characteristics.** (**A**, **B**) Heatmaps showed the expression levels of different m6A regulatory genes in low-risk and high-risk patients. The distribution of clinicopathological features in low-risk and high-risk groups was compared. (**C**) ROC curve shows the signature of risk prediction efficiency in endocrine system tumors.

In our analysis of the tumors, we found that there were significant differences in survival status, T status and who grade between the high-risk group of ACC (Figure 4A) and the low-risk group, and significant differences in survival status between the high-risk group and the low-risk group of PCPG (Figure 4A), THCA (Figure 4A) and THYM (Figure 4A). Besides, we observed that the OS of high-risk patients in each disease dataset was much shorter than that of low-score patients.

We also analyzed the expression of six selected m6A RNA methylation regulators in the “Total 1” dataset in high-risk and low-risk patients. We observed significant differences in “Total 1” (Figure 4B) between the high-risk group and the low-risk group, and the high-risk group associated with lower survival status (P < 0.01), higher who grade (P < 0.01), greater T (P < 0.01), and more lymph node metastasis (P < 0.01). We then analyzed the “Total 2” dataset (Figure 4B) and failed to find the difference between the high-risk group and the low-risk group in the clinical information of gender and survival status. But OS of the high-risk patients in both “Total” datasets was much shorter than the OS of the low-score patients.

ROC curves showed that the risk score was a perfect predictor of 5-year survival in ACC patients (AUC = 80.1%) (Figure 4C), PCPG (AUC = 88.8%) (Figure 4C), THCA (AUC =72.8%) (Figure 4C) and THYM (AUC = 81.4%) (Figure 4C), “Total 1” (AUC =68.3%) (Figure 4C), and “Total 1” (AUC =70.9%) (Figure 4C).

We then performed univariate and multivariate Cox regression analyses for the dataset to determine whether the risk characteristics were independent predictors. Univariate regression analysis of ACC data showed that risk scores, who rating and T status were all correlated with OS (P <0.01, Table 1). When these factors were included in multivariate Cox regression, T status (P <0.01) and risk scores (P =0.01) remained significantly correlated with OS (Table 1). Univariate regression analysis of PCPG data showed that the risk score was correlated with OS (P =0.004, Table 1). When these factors were included in the multivariate Cox regression, the risk scores (P =0.002) remained significantly correlated with OS (Table 1). Univariate regression analysis of THCA data showed that risk scores (P =0.014), grade (P =0.003), T status (P =0.028), and age (P <0.01) were all associated with OS (Table 1). When these factors were included in multivariate Cox regression, age (P <0.01) remained significantly correlated with OS (Table 1). A univariate regression analysis of THYM data shows that the risk score (P <0.01) is OS related. In “Total 1”, risk scores (P <0.001), who rating (P <0.001), and T status (P <0.001) were all associated with OS by univariate analysis (Table 1). When these factors were included in the multivariate Cox regression, the risk scores (P <0.001), who grade (P =0.002), and T status (P =0.012) remained significantly correlated with OS (Table 1). In “Total 2”, the risk scores (P =0.012) was correlated with OS by univariate analysis (Table 1). When these factors were included in the multivariate Cox regression, the risk scores (P =0.013) remained significantly correlated with OS (Table 1). In general, risk scores could be used to greatly predict the prognosis of patients with endocrine system tumors. Gender was not associated with the prognosis of endocrine tumors.

**Table 1:** The univariate and multivariate COX regression analysis of m6A regulatory genes for patients' overall survival (OS) in endocrine system tumors.

**Cancer types**		**Univariate**		**Multivariate**	
		**HR (95%CI)**	**P**	**HR**	**P**
ACC	gender	1.100591489	0.814544864	1.038052354	0.930365584
	stage	2.760634449	<0.01	0.732881514	0.571016476
	T	3.237383176	<0.01	3.747329892	0.005749074
	N	1.829492414	0.269274473	2.918855029	0.137599231
	riskScore	1.193940656	<0.01	1.13395091	0.010312723
PCPG	gender	3.131482468	0.198106512	10.54784495	0.0623891
	riskScore	1.001445171	0.004298715	1.002063944	0.001882145
THCA	age	1.155357049	<0.01	1.166359177	0.000826538
	gender	0.868700107	0.860752257	0.731485506	0.748013828
	stage	2.729834716	0.002868951	0.796474584	0.823787006
	T	2.4428418	0.028302365	2.563634273	0.295472549
	M	2.560644338	0.379583475	3.906592763	0.292240082
	N	2.086807866	0.298770285	0.388357974	0.367125281
	riskScore	1.020547508	0.014232509	1.015493346	0.147944117
THYM	gender	0.629466902	0.49190441	0.66426715	0.550430644
	riskScore	<0.01	0.000837672	>1	0.000987518
Total1	gender	1.596802381	0.15281085	1.342885697	0.38062215
	stage	2.73244358	<0.01	2.124943581	0.002348671
	T	3.351611782	<0.01	1.668245363	0.051414123
	N	0.616791825	0.152388789	0.400896738	0.011567273
	riskScore	164.1049178	<0.01	47.82932365	<0.01
Total2	gender	1.271577618	0.642975417	1.180127876	0.749370968
	riskScore	414.944273	0.012117844	407.8618477	0.01288766

Note: Ambiguous variables (Nx, Mx, N/A, discrepancy and Gx) were excluded. ACC, Adrenal cortical carcinoma;

PCPG, Pheochromocytoma and Paraganglioma; THCA, Thyroid carcinoma; THYM, Thymoma;

Total1, Adrenal cortical carcinoma and Thyroid carcinoma; Total2, Pheochromocytoma and Paraganglioma and Thymoma.

## DISCUSSION

M6A regulatory genes have been found to be associated with malignant transformation and development of tumors [6], but its role in endocrine tumors remains unclear. Due to the heterogeneity of each tumor, we found that in four tumors, only a few m6A regulatory genes showed no difference in the expression of this gene, while most m6A regulatory genes showed differences in the expression of endocrine system tumors compared with normal tissues (Figure 1). All above prove that the expression of regulatory factors of m6A regulatory genes is closely related to endocrine system tumors.

Studies have shown that the core proteins of the m6A methyltransferase do not exist in isolation, they can function by forming complexes [29]. For example, WTAP plays an important role in recruiting METTL3 and METTL14 [30]. A study of Ries et al. showed that m6A modification can promote the co-occurrence and separation of mRNA and binding protein YTHDF protein, and the separation from YTHDF protein can in turn regulate the localization and translation efficiency of mRNA [31]. We then investigated the expression correlation of the m6A regulatory genes. It was found that there was a positive correlation between WTAP and METTL3 expression in each dataset. When the WTAP gene is up-regulated, the METTL3 gene may also be up-regulated. There was an article to explain the effect of METTL3 on WTAP, and the upregulation of METTL3 can lead to an increase of the WTAP protein level in leukemia, causing the carcinogenic effect of leukemia [32]. In addition to the positive correlation between ALKBH5 and FTO expression in THYM (Figure 1B), ALKBH5 showed a negative correlation with other genes. Some articles have demonstrated the role of ALKBH5 in various cancers, such as pancreatic cancer [33], breast cancer [34], and gastric cancer [35]. However, ALKBH5 in colorectal cancer [36] plays an anti-cancer effect. These data also illustrate the interaction of gene expression level between m6A regulatory genes.

It is showed that the cluster 1 subgroup (Figure 2D) was significantly correlated with better survival status (P < 0.01), higher who grade (P < 0.01), and higher T status (P < 0.01). The cluster 2 subgroup was associated with worse survival status, lower who grade (P < 0.01), and lower T degree (P < 0.01). In addition, we observed that the overall survival rate (OS) of the cluster 1 subgroup was significantly shorter than that of the cluster 2 subgroup (Figure 2D). We then clustered the other tumors in the same way, observed some similar results and other differences. According to the THCA dataset, we observed no significant difference in OS (Figure 2D) between the cluster 1 subgroup and the cluster 2 subgroup, and it may be necessary to observe the survival in patients with the disease for a longer time. Besides, if only from the aspects of living condition and gender analysis PCPG and THYM datasets (Figure 2B, 2E), we found no significant difference of clinical features.

Then we conducted cluster analysis based on the correlation of expression of m6A regulatory genes in endocrine system tumors, and analyzed ACC (Figure 2D) findings that cluster 1 subgroup was significantly correlated with poor survival status (P < 0.01), higher who grade (P < 0.01), higher T degree (P < 0.01) and poor prognosis. The cluster 1 subgroup of THCA (Figure 2D) was found to be correlated with higher who grade (P < 0.01) and higher T degree (P < 0.01). It may still take a long follow-up to find the difference in OS in THCA dataset. We observed that 'Total 1” (dataset of ACC and THCA) found that its cluster 1 subgroup (Figure 2D) was associated with poorer survival status (P < 0.01), higher who grade (P < 0.01), greater T degree (P < 0.01), more lymph node metastasis (P < 0.01) and poorer prognosis. The above conclusions are sufficient to indicate that m6A regulatory genes are closely related to the clinicopathological characteristics of endocrine system tumors.

A study found that the m6A regulatory gene not only causes tumor progression, but also leads to tumor suppression [37]. METTL14 is an anti-metastasis factor in primary Hepatocellular carcinoma (HCC), and down-regulation of METTL14 suggests poor prognosis in HCC patients and leads to HCC progression and metastasis [38]. However, METTL14 plays a carcinogenic role in endometrial cancer by regulating AKT activation through m6A modifications [39]. According to the gene expression and its clinical factors, we observed that METTL14 is the protective gene in ACC dataset (Figure 4A). The latest literature shows that IGF2BP plays an important role in the development of tumors [40]. We found that IGF2BP3 is closely related to the prognosis of PCPG (Figure 4A), and is risk gene. It has been reported that YTHDF3 plays an important role in m(6) A-modified transcripts [41], and FTO is also extremely important for tumorigenesis [42]. It is showed that YTHDF3 and FTO are closely related to the prognosis of THCA (Figure 4A), and they are all risk genes. We found that WTAP is protective gene which is closely related to the prognosis of THYM (Figure 4A), “Total 1” data (Figure 4B), and “Total 2” (Figure 4B). Current literature has shown that WTAP plays an important role in inhibiting cancer in gastric cancer [43], ovarian cancer [44], and bladder cancer [45], which is different from the results of this paper, possibly due to tumor heterogeneity. We obtained m6A regulatory genes closely related to OS through the analysis of “Total 1” data (Figure 4B), including METTL14, WTAP, RBM15B, METTL16, FTO and IGF2BP1. Among them, IGF2BP1 is a risk gene. In “Total 2” (Figure 4B), WTAP and METTL16 have a significant correlation with OS, and they are all protective genes. In general, it can be considered that WTAP, METTL16 and IGF2BP3 play important roles in the development of endocrine system tumors, WTAP, METTL16 are protective genes, IGF2BP3 is danger genes.

We also conducted risk assessment, univariate and multivariate Cox regression analysis, and T status (P <0.01) and risk scores (P =0.01) were significantly correlated with OS of ACC (Table 1). The risk scores (P =0.002) remained significantly correlated with OS of PCGA (Table 1). The risk scores remained significantly correlated with OS of THYM (Table 1). Age (P <0.01) was significantly correlated with the OS of THCA (Table 1). In “Total 1” (Table 1), the risk scores (P <0.001), grade (P =0.002), and T status (P =0.012) remained significantly correlated with OS. In “Total 2” (Table 1), the risk scores (P =0.013) remained significantly correlated with OS. In general, we found that gender was not associated with the prognosis of endocrine tumors, and risk scores could be used to greatly predict the prognosis of patients with endocrine system tumors.

In conclusion, our findings systematically demonstrate that m6A regulatory genes expression is closely related to endocrine system tumors, though the expressions of m6A regulatory genes are heterogeneous in the endocrine system tumors. We not only analyzed the correlation between tumors and m6A regulatory genes, but also studied the common characteristics between tumors and m6A regulatory genes in endocrine system tumors, analyzed the clinical prognostic factors of m6A regulatory genes based on its interactivity and expression characteristics. We observed that WTAP, METTL16 and IGF2BP3 play important roles in the development of endocrine system tumors, WTAP, METTL16 are protective genes, IGF2BP3 is danger genes. We also found that gender was not relative with the prognosis of endocrine tumors, and risk score is a prognosis prediction of patients with endocrine system tumors. In summary, WTAP, METTL16 and IGF2BP3 can be regarded as a new promising biomarker, and risk score can be used for the prognosis of UM and the formulation of treatment strategies.

## MATERIALS AND METHODS

### Data sources

RNA-seq transcriptome data and the corresponding clinicopathological and prognostic information were obtained for 79 ACC patients,183 PCPG patients, 510 THCA patients, 119 THYM patients from the TCGA database (https://cancergenome.nih.gov/). And TCGA normal group data were obtained for 0 ACC patients, 3 PCPG patients, 58 THCA patients, 2 THYM patients. Since the number of TCGA controls was insufficient, we combined the Genotype-Tissue Expression (GTEx) (http://www.bio-info-trainee.com/3705.html) database with a small number of TCGA normal group data to form the control group. We added GTEx_adrenal gland to the ACC control group (n=128), added GTEx_adrenal gland to the PCPG control group (n=128), added GTEx_Thyroid to the THCA control group (n=279), and added GTEx_Heart (Atrial Appendage, Left Ventricle) to the THYM control group (n=377) (Table 2).

**Table 2:** RNA-seq dataset of tumor group and control group in this study.

**Cancer types**	**Tumor count**	**Normal count**	**Amount**
ACC	TCGA_ACC 79	GTEx_adrenal gland 128	207
PCPG	TCGA_PCPG 183	TCGA_PCPG 3 + GTEx_adrenal gland 128 = 131	314
THCA	TCGA_THCA 510	TCGA_THCA 58 + GTEx_Thyroid 279 = 337	847
THYM	TCGA_THYM 119	TCGA_THYM 2 + GTEx_Heart (Atrial Appendage, Left Ventricle) 377 = 379	498
Total 1	TCGA_ACC 79+ TCGA_THCA 510=589	GTEx_adrenal gland 128+ TCGA_THCA 58 + GTEx_Thyroid 279 = 465	1054
Total 2	TCGA_PCPG 183+ TCGA_THYM 119=302	TCGA_PCPG 3 + GTEx_adrenal gland 128+ TCGA_THYM 2 + GTEx_Heart (Atrial Appendage, Left Ventricle) 377 =510	812

Note: ACC, Adrenal cortical carcinoma; PCPG, Pheochromocytoma and Paraganglioma; THCA, Thyroid carcinoma; THYM, Thymoma; Total 1, Adrenal cortical carcinoma and Thyroid carcinoma; Total 2, Pheochromocytoma and Paraganglioma and Thymoma.

**Figure 5:**
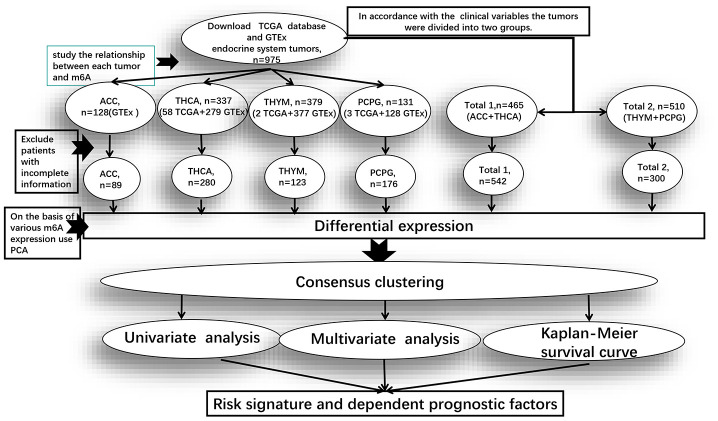
**Clinical data processing and subsequent studies in the pan-oncogenic analysis of m6A regulatory genes in endocrine system tumors.** Total 1 is the dataset of ACC and THCA, and Total 2 is the dataset of PCPG and THYM. The figure shows from TCGA and GTEx after downloading data in the database of datasets, Exclude patients with incomplete information after the rest of the sample size and the process of analysis of m6A regulatory genes.

In order to study the common characteristics of the expression value of m6A regulatory genes between endocrine system tumors, we divided the four groups of tumors for different clinical variables into two groups. “Total 1” is the dataset of ACC and THCA, mainly analyzing the clinical information of their common survival time, survival status, gender, grade, tumors range and lymph node metastasis. “Total 2” is the dataset of PCPG and THYM, mainly analyzing the only clinical information of survival time, survival status and gender (Figure 5).

### Selection of m6A regulatory genes

We compiled a list of 23 tumor-related m6A regulatory genes [46–49] based on published literature, and then restricted the list to the genes with available RNA expression data in TCGA data. We obtained mRNA expression data of 23 m6A-related genes [50] from the TCGA database and compared the relationship between the expression of 23 m6A-related genes and the clinicopathological variables of endocrine system tumors.

### Bioinformatic analysis

We used the “R” package (R v3.6.2) for analysis (Figure 5). And the Practical Extraction and Report Language (Perl) was used to accurately handle text format that require R package analysis. Firstly, we used the “limma” package to conduct gene difference analysis, used the “pheatmap” and the “vioplot” packages to visualize the expression of 23 genes, and then used the “corrplot” package to analyze the correlation of each gene expression in the tumors. Due to the expression similarity, the “ConsensusClusterPlus” package was used to cluster endocrine system tumors into different groups, and the “ggplot2” package was used to analyze the gene expression of each cluster group. Moreover, we removed patients with incomplete clinical information and used the “survival” package to determine the survival prognosis, and used the “pheatmap” package to visualize the difference expression of 23 genes between cluster 2 and cluster 1.

Next, we used the “forestplot” package to construct univariate COX proportional regression model, and evaluated different m6A expression values and used the “glmnet” package to obtain m6A regulatory genes. Moreover, we use the “survival” package and the “survivalROC” package to analyze the survival of the cluster, and used the “pheatmap” package to visualize the relationship between the expression of relative genes and the different clinical factors. Finally, we used the “forestplot” and the “survival” packages to screen out m6A regulatory genes and clinical factors with a significant influence on prognosis.

### Statistical analysis

In order to determine m6A RNA methylation regulating factors in the prognostic role of endocrine tumor patients, we use the Cox univariate analysis data from the TCGA and GTEx database. From this, we selected value genes significantly associated with survival (P < 0.05), which we chose for further analysis of the function and developed a potential risk signature with the LASSO Cox regression algorithm [51]. Risk score calculation is as follows:

$$\text{Risk score }=\overset{n}{\underset{i=1}{\sum }}{\text{Coef}}_{i}\times {\text{ x}}_{i}$$

where Coef_i_ is the coefficient, and x_i_ is the expression value of each selected gene. This formula was used to calculate the risk scores for each patient.
